# Complete Genome Sequence of a Virulent African Swine Fever Virus from a Domestic Pig in Ukraine

**DOI:** 10.1128/MRA.00883-19

**Published:** 2019-10-17

**Authors:** Ganna Kovalenko, Anne-Lise Ducluzeau, Liudmyla Ishchenko, Mykola Sushko, Maryna Sapachova, Nataliia Rudova, Oleksii Solodiankin, Anton Gerilovych, Ralf Dagdag, Matthew Redlinger, Maksym Bezymennyi, Maciej Frant, Christian E. Lange, Inna Dubchak, Andrii A. Mezhenskyi, Serhiy Nychyk, Eric Bortz, Devin M. Drown

**Affiliations:** a Institute of Veterinary Medicine (IVM), National Academy of Agrarian Sciences of Ukraine, Kiev, Ukraine; b Institute of Arctic Biology, University of Alaska Fairbanks, Fairbanks, Alaska, USA; c State Scientific Research Institute of Laboratory Diagnostics and Veterinary and Sanitary Expertise (SSRILDVSE), Kiev, Ukraine; d National Scientific Center Institute of Experimental and Clinical Veterinary Medicine (NSC IECVM), Kharkiv, Ukraine; e Department of Biological Sciences, University of Alaska Anchorage, Anchorage, Alaska, USA; f National Veterinary Research Institute (NVRI), Pulawy, Poland; g Metabiota, San Francisco, California, USA; h Joint Genome Institute, Lawrence Berkeley National Laboratory, Berkeley, California, USA; i Department of Biology and Wildlife, University of Alaska Fairbanks, Fairbanks, Alaska, USA; KU Leuven

## Abstract

Here, we report the complete genome sequence of an African swine fever (ASF) virus (ASFV/Kyiv/2016/131) isolated from the spleen of a domestic pig in Ukraine with a lethal case of African swine fever. Using only long-read Nanopore sequences, we assembled a full-length genome of 191,911 base pairs in a single contig.

## ANNOUNCEMENT

African swine fever (ASF) is a hemorrhagic viral disease of pigs that is characterized by high mortality and significant economic losses in domestic pigs. The virulent p72 genotype II lineage of African swine fever virus (ASFV; Asfivirus, family Asfarviridae) has been spreading rapidly from Georgia to Eastern and Central Europe since 2007 (1, 2). The global situation dramatically escalated, with confirmation that ASFV reached China in 2018 and had spread quickly across East Asia in 2019 (2, 3). In Ukraine, 485 laboratory-confirmed outbreaks of ASFV were recorded between 2012 and 2019 (4). The continued detection of outbreaks and the spread over the entire country raises concerns of ASFV becoming endemic in Ukraine.

Tissue samples were collected from a domestic pig from ASF outbreak number 131 in Kyiv Oblast, Ukraine, in 2016. The samples were frozen, and total DNA was extracted in duplicate from spleen tissue using the PowerMicrobiome RNA isolation kit (Mo Bio) following the manufacturer’s protocol. The extraction kit retains both RNA and DNA, and a diagnostic conventional real-time PCR (RT-PCR) (LSI VetMax p72 PCR kit; Thermo Fisher Scientific) confirmed ASFV in the sample (http://www.asf.vet.ua/index.php/asfinukraine).

For full-genome sequencing, we purified the extracted DNA using Agencourt AMPure XP beads (Beckman Coulter) with three different bead-to-DNA (vol/vol) ratios (0.4×, 0.7×, and 1.0×) to retain a range of fragment lengths. We collected DNA sequence data across two sequencing runs using the Oxford Nanopore Technologies (ONT) MinION platform (Table 1). For sequencing run 1, we pooled 522 ng of DNA, including 300 ng of DNA purified from the 0.4× cleanup and 222 ng from the 0.7× cleanup. For sequencing run 2, we used 490 ng of DNA from the 1.0× cleanup. For each run, we prepared a rapid sequencing library (SQK-RAD004; ONT) and sequenced the prepared library on an R9.4.1 flow cell (FLO-MIN106) for 48 h using a MinION Mk1B device. We base called raw data using Guppy v3.1.5 (ONT) with a high-accuracy model (dna_r9.4.1_450bps_hac.cfg) and default parameters. Before assembly, we created a quality-controlled data set using Porechop v0.2.4 (https://github.com/rrwick/Porechop) with default parameters to trim adaptors and discard sequences with middle adapters (-discard_middle) and Filtlong v0.2.0 (https://github.com/rrwick/Filtlong) to filter by a quality (Q) score of ≥10 (-min_mean_q 90).

**TABLE 1:** Summary of sequencing data statistics

Data set	No. of reads	Yield (bp)	Avg length (bp)	Avg quality (Q score)
Run 1 raw	2,213,244	5,213,092,675	2,355.4	10.5
Run 1 quality controlled	1,649,561	3,994,767,329	2,421.7	13.1
Run 2 raw	3,290,571	6,235,197,650	1,894.9	10.5
Run 2 quality controlled	2,430,267	4,631,455,885	1,905.7	13.1
Total quality controlled	4,079,828	8,626,223,214	2,114.4	13.1
After *S. scrofa* removed	98,078	27,426,602	279.6	12.5

To remove reads likely originating from the host, we used Minimap2 v2.17-r941 (4) with default parameters for Nanopore reads (-ax map-ont) to align our quality-controlled data to the Sus scrofa 11 reference genome (GenBank assembly accession no. GCA_000003025). We extracted the unmapped reads for *de novo* assembly using SAMtools v1.9 (5). We assembled the genome using Flye v2.4.2 (6) with default parameters specifying the estimated genome size (-genome-size = 200k) and Nanopore reads (-nano-raw). Our raw assembly (coverage, 32×) consists of 227,741 bp in 9 linear contigs (*N*_50_, 193,207 bp). We confirmed via an NCBI blastn search (7) that only the longest contig, 193,207 bp, was ASFV. The top hits for this contig had a greater than 99% identity to recently published ASFV genomes (e.g., GenBank accession no. LR536725 and MK128995). For this longest contig, we used a polishing pipeline specific to the error profile of Nanopore reads. We completed two rounds of polishing with the graphics processing unit (GPU)-enabled version of Racon v1.3.3 (https://github.com/clara-genomics/racon-gpu) (8) with the following parameters: score for matching bases (-match 8), score for mismatching bases (-mismatch -6), threshold for average base quality of windows (-quality-threshold -1), default gap penalty (-gap -8), default window (-window-length 500), and number of Compute Unified Device Architecture (CUDA) batches (-c 2). We completed a final round of Nanopore-specific polishing with Medaka v0.8.0 (https://github.com/nanoporetech/medaka).

Our polished assembly (coverage, 27×) consists of 191,911 bp in a single linear contig (GC content, 38.3%). We confirmed that this is a Georgia lineage p72 genotype II strain by using blastn (7) to compare the sequence variation at the p72 (B646L) gene and found 99.9% identity to Georgia 2007/1 (GenBank accession no. FR682468), with the only difference associated with two different homopolymer regions. We used MAFFT v7.388 (9, 10) to align our polished assembly with the Georgia 2007/1 assembly. A visual inspection of this alignment with Geneious Prime v 2019.1.1 confirmed overlap of the complete genome, including ends. We found a 10-nucleotide insertion (GGAATATATA) between the I73R and the I329L genes, as previously reported (11–13). According to those previous reports, this insertion is not linked to attenuation or virulence. This insertion is present in the China/2018/AnhuiXCGQ genome (GenBank accession no. MK128995) but absent from ASFV/POL/2015/Podlaskie (accession no. MH681419) and Georgia 2007/1. We did not detect the left-end genome deletion found in an attenuated strain from Estonia (14). This is the first full-genome report of a virulent Georgia lineage p72 genotype II strain from the multiyear zoonotic outbreaks of ASF in Ukraine, highlighting the accuracy and deployability of Nanopore sequencing on a MinION platform for analysis of an emerging disease that has spread across Eurasia.

### Data availability.

This genome sequence has been deposited in GenBank under the accession no. MN194591. The version described in this paper is the first version, MN194591.1. Raw data for this project can be found in the GenBank SRA under accession no. PRJNA555080.
